# Detection of Gas6/AXL complex and its expression changes in patients with ST-segment elevation myocardial infarction

**DOI:** 10.3389/fmed.2025.1653708

**Published:** 2025-08-26

**Authors:** Zhao Yin, Xiaohua Nie, Tianlun Li, Bei Zhao, Shouli Wang, Liguo Zhang, Xuyuan Zhang, Zhaoliang Shan

**Affiliations:** ^1^Postgraduate School, Medical School of Chinese PLA, Beijing, China; ^2^Department of Cardiology, The Sixth Medical Center, Chinese PLA General Hospital, Beijing, China; ^3^Department of Cardiology, The Ninth Medical Center of Chinese PLA General Hospital, Beijing, China; ^4^National Laboratory of Biomacromolecules, Institute of Biophysics, Chinese Academy of Sciences, Beijing, China

**Keywords:** Gas6/AXL complex, AXL, monoclonal antibody, flow cytometry, ELISA

## Abstract

**Introduction:**

It has been reported Gas6/AXL pathway participates in several diseases. However, due of the lack of specific antibody, the significance and change circulatory Gas6/AXL complex in related diseases remain unclear.

**Methods:**

The mice were immunized with human AXL protein which were expressed by L cells to obtain monoclonal antibodies (mAbs). The flow cytometry was used to monoclonal antibodies screening and binding region mapping. To prepare an AXL-Gas6 specific mAb coated ELISA plate, and test the level of AXL-Gas6 complex in human blood.

**Results and discussion:**

We successfully obtained an AXL monoclonal antibody only binding to Gas6/AXL complex. Using the antibody, we firstly found that AXL expressed on human pDCs had bind to Gas6. Not only applicated in flow cytometry, this novel antibody could also be used as capture antibody in ELISA. Depend on this antibody, we firstly discovered that sAXL Gas6 complex was significantly increased in patients with AMI. Compared to AXL detected by commercial kit, AXL-Gas6 complex we measured emerged as a more sensitive biomarker and risk factor for patients with ST-segment elevation myocardial infarction.

## Introduction

AXL is a member of TAM receptor family, which includes three members: Tryo3, AXL and MerTK (1). It is well-established that AXL is expressed on various cell types, including myocardial cells (2). AXL binds to its ligand Gas6 and is involved in hypertension (3), cardiac fibroblasts (4) and platelet activation (5). The activation of Gas6-AXL signaling has been showed to provide protection against ischemia–reperfusion injury (6) and sepsis induced injury (7). AXL, which is expressed on the cell membrane, can be cleaved to form soluble AXL, a process dependent on the activity of a disintegrin and metalloproteinase10 (ADAM10) and ADAM17 (8).

Soluble AXL (sAXL) has garnered significant attention in the field of cardiovascular diseases (CVD) as a biomarker in recent years. Research indicates that sAXL holds substantial potential for predicting the prognosis and mortality in patients with CVD, particularly within specific patient populations. Studies have demonstrated that sAXL exhibits considerable predictive value in patients with heart failure with preserved ejection fraction (HFpEF), reliably predicting both all-cause and cardiovascular mortality in these individuals (9). Moreover, in those with acute coronary syndrome (ACS), sAXL levels are significantly elevated compared to healthy controls and show a positive correlation with inflammatory markers such as high-sensitivity C-reactive protein (hs-CRP) and tumor necrosis factor-alpha (TNF-*α*) (10). This finding suggests that sAXL may play a crucial role in the inflammatory response and cardiovascular events in ACS, with elevated levels potentially indicating a poor prognosis.

Soluble AXL in human blood exists in two forms: free sAXL and the Gas6/AXL complex, depending on its binding to Gas6 (11). These different forms of sAXL may indicate varying pathophysiological states (12). Under normal conditions, there is an excess of sAXL in serum, with Gas6 serving as the limiting factor for complex formation (11). In patients with chronic limb ischemia (CLI), sAXL levels are significantly increased, and both sAXL and its ligand Gas6 concentrations are higher compared to the control group without atherosclerotic symptoms (13). However, in sepsis, the concentration of sAXL increases in patients, while the concentration of Gas6 remains relatively unchanged (14). This discrepancy between sAXL and Gas6 suggests that the level of the Gas6/AXL complex may vary correspondingly. Recently, sAXL has been identified as a novel biomarker in both heart failure (2, 15) and acute coronary syndrome (10). Furthermore, sAXL levels in ST-segment elevation myocardial infarction (STEMI) have been associated with cardiac function and left ventricular remodeling (16). However, the dynamics of the two different forms of sAXL, particularly the Gas6/AXL complex, in CVD remains unclear.

Despite this, there is a notable scarcity of discussion regarding the Gas6/AXL complex in the existing literature. To date, reliable and direct methods for accurately detecting the Gas6/AXL complex in human plasma remain unavailable. Consequently, the implications of variations in the levels of the Gas6/AXL complex in CVD, such as acute myocardial infarction (AMI), remain largely unexplored. Therefore, in the current study, we developed a monoclonal antibody (αAXL-4#) that recognizes the Gas6/AXL complex and can be employed as a capture antibody. Using αAXL-4#, we established an ELISA detection assay for the detection of the Gas6/AXL complex and preliminarily explored its clinical significance in AMI.

## Materials and methods

### Cloning and expression of AXL and Gas6

The coding sequences of human AXL (hAXL), mouse AXL (mAXL), and hAXL truncations were cloned into an expression vector under EF1A promotor and connected to the GFP reporter via an IRES sequence.

To anchor Gas6 to the cell membrane, the coding sequence of hGas6 was cloned into the pDisplay Mammalian Expression Vector, which included a transmembrane domain.

To facilitate the expression and purification of AXL and Gas6, the hGas6, mGas6 or hAXL extracellular domain (ECD) with 6xHis-tag, were cloned into the pTT3 vector. The proteins were produced in 293F Human Embryonic Kidney cells and purified with a protein A agarose prepacked column.

### Antibody generation

In this study, L929 cells (ATCC® CRL-2648™) were transfected with recombinant plasmids containing the hAXL gene, designated as L-hAXL. Prior to immunization, L-hAXL cells were incubated with Gas6-his fusion protein for 20 min. Subsequently, BALB/c mice were immunized monthly using either the L-hAXL cells or the cell-protein mixture combined with CpG1826 (InvivoGen) as an adjuvant. Sera from immunized mice were analyzed via flow cytometry for reactivity towards hAXL-expressing HEK293T cells (293-hAXL) with or without GAS6-his. Hybridoma formation was conducted following our previously established protocols (17). Briefly, splenocytes were isolated from the immunized mice and fused with SP2/0 cells to generate hybridoma cells. The supernatant from these hybridoma cells was collected and added to 96-well plates containing 293-hAXL, which had been pre-treated with or without Gas6-his for 30 min at 4°C. After washing with PBS containing 2% FBS and 2 mM EDTA twice, the cells were stained with PE-goat anti-mouse IgG (BioLegend, Poly4053, 405307) and incubated away from light for 30 min at 4°C. Both GFP and PE positive wells were selected using an Attune NxT Flow Cytometer (Thermo Fisher Scientific), and monoclonal cells capable of stably producing AXL antibodies were screened. AXL antibodies were purified with a protein A agarose prepacked column. The AXL antibody 4# recognizing hAXL and hGAS6 was predicted by AlphaFold (18).

### Binding region mapping

To analyze the binding region the AXL antibodies, a series of AXL truncations were generated. Only express the first immunoglobulin-like (Ig-like) domain (Domain 1), only express the second Ig-like domain (Domain 2), only express the first fibronectin type III (FNIII) domain (Domain 3), only express the second FNIII domain (Domain 4), only missing the first Ig-like domain (Domain 234), only missing the second Ig-like domain (Domain 134), only missing the first FNIII domain (Domain 124), only missing the second FNIII domain (Domain 123). Antibodies were analyzed via flow cytometry for reactivity towards hAXL or hAXL truncations, both in the presence and absence of GAS6-his.

### Flow cytometry

To further validate the necessity of Gas6 for AXL antibodies, 293-hAXL cells were plated in round 96-well plates and treated with varying concentrations of Gas6-his fusion protein. Subsequently, these cells were co-cultured with either AXL antibodies or an isotype control for 30 min at 4°C. Following this, the cells were stained with PE-goat anti-mouse IgG following two washes. The mean fluorescence intensity (MFI) of PE-positive cells was measured using an Attune NxT Flow Cytometer (Thermo Fisher Scientific). Additionally, HEK-293T cells were transfected with the pDisplay-hGAS6 vector, designated as 293-hGas6, which anchors Gas6 to the cell membrane. Antibodies were analyzed via flow cytometry for their reactivity towards 293-hGas6, both in the presence and absence of AXL-his.

To analyze the binding specificity of AXL antibodies across different species, the reactivity of antibodies was assessed using flow cytometry against 293-hAXL and 293-mAXL, both in the presence and absence of hGas6-his or mGas6-his.

To analyze the binding ability, AXL antibodies were incubated with cells from various sources for 30 min at 4°C. Subsequently, the cells were stained with PE-goat anti-mouse IgG after undergoing two washing steps. The MFI of the PE-positive cells was measured using either an Attune NxT Flow Cytometer (Thermo Fisher Scientific) or an LSRFortessa (BD).

### Formation of the Gas6/AXL complex and enzyme-linked immunosorbent assay (ELISA)

As previously reported (11), mix Gas6 (250 ng/mL, HY-P70470, MCE) with AXL (30 ng/mL, DY154, R&D Systems) in a total volume at 20 μL for 1 h to obtain the AXL-Gas6 complex. The mixture was further diluted to the required concentrations using PBS containing 0.05% Tween and 0.1% bovine serum albumin (BSA). Sandwich ELISA was employed for the quantitative determination of the sAXL-Gas6 complex, AXL and Gas6. Generally, plates were coated with αAXL-4# antibody or AF154 (DY154, commercial kit from R&D system) or Gas6 antibody (DY885B, commercial kit from R&D Systems) overnight at 4°C, blocked with PBS containing 0.05% Tween and 0.1% BSA for 1 h at room temperature, and then incubated with plasma or standard protein (AXL-Gas6 complex, AXL protein or Gas6 protein from commercial kit) for 2 h at room temperature. Detection antibody, streptavidin-HRP, substrate solution and stop solution were added in sequence according to the instructions provided by R&D system. Between each step, plates were washed with PBS containing 0.05% Tween 3–5 times. A microplate reader (BioTek) was used to determine the optical density (OD) at 450 nm.

### Patients and clinical characteristics

A total of 64 AMI patients who underwent primary or emergency percutaneous coronary intervention (PCI) treatment in our hospital from October 2014 to December 2016 were selected (excluding those who did not successfully receive PCI after thrombolysis, those with surgical contraindications, and those with incomplete clinical and laboratory data), including 40 patients with STEMI and 24 patients with NSTEMI. Twenty-four patients with chest pain or shortness of breath in the department of cardiology in the same period were selected as the control group. The baseline data and clinical characteristic were recorded (Table 1). The retrospective study complied with the ethical guidelines of the 1975 Declaration of Helsinki, and was approved by the ethics committee of The Ninth Medical Center of Chinese PLA General Hospital.

**Table 1:** The baseline data and clinical characteristic.

Characteristic	STEMI *N* = 40	NSTEMI *N* = 24		Normal *N* = 24	*p-*value
Cardiovascular risk factor					
Age/year, x ± s	57.63 ± 13.01	60.54 ± 12.99		59.21 ± 9.08	0.637
Male, *n* (%)	31 (77.50)	18 (75.00)		15 (62.50)	0.059
BMI/(kg·m^−2^), x ± s	25.76 ± 3.07	24.34 ± 2.87		25.21 ± 4.01	0.254
Hyperlipemia, *n* (%)	28 (70.00) ^*^	10 (41.70) ^*^		2 (8.30)	<0.001
Smoking, *n* (%)	24 (60.00)	11 (45.80)		7 (29.20)	0.056
Diabetes mellitus, *n* (%)	9 (22.50)	10 (41.70)		9 (37.50)	0.220
Hypertension, *n* (%)	19 (47.50)	14 (58.30)		9 (37.50)	0.352
Family history of CAD, *n* (%)	15 (38.50)	7 (31.80)		11 (45.80)	0.621
Cardiac function					
LVEF/%, x ± s	54.13 ± 6.06 ^*^	57.83 ± 8.10	62.43 ± 5.32	<0.001	
BNP/(pg·mL^−1^), M (QL, QU)	198.00 (79.20, 328.50) ^*^	518.00(261.50, 1813.00) ^*^	66.30 (40.05, 111.00)	<0.001	
Biomarkers					
FPG/(mmol·L^−1^), x ± s	9.06 ± 4.21 ^*^	7.55 ± 2.99	5.34 ± 1.48	<0.001	
HDL-C/(mmol·L^−1^), x ± s	1.36 ± 0.23	1.24 ± 0.27 ^*^	1.49 ± 0.27	0.004	
LDL-C/(mmol·L^−1^), x ± s	2.83 ± 0.99	2.41 ± 0.76 ^*^	2.21 ± 0.55	0.012	
hs-CRP/(mg·L^−1^), M (QL, QU)	1.45 (0.42, 2.87)	3.26 (1.12, 10.27) ^*^	1.14 (0.14, 1.68)	0.021	
HbA1c/%, x ± s	6.56 ± 1.67	7.10 ± 1.98	6.04 ± 1.08	0.095	
TC/(mmol·L^−1^), x ± s	4.75 ± 1.27	4.34 ± 1.02	4.33 ± 0.72	0.213	
TG/(mmol·L^−1^), M (QL, QU)	1.10 (0.75, 1.70)	1.40 (0.90, 1.80)	0.95 (0.80, 1.50)	0.548	
Uric acid/(μmol·L^−1^), x ± s	328.10 ± 131.97	332.29 ± 110.42	353.58 ± 111.22	0.707	

### Statistics

Statistical analyses were performed using STATA. Continuous variables were analyzed by using two-tailed paired Student *t*-test or one-way ANOVA following by Dunnett’s multiple comparison test. Categorical variables were analyzed by using the and Chi square test. In Logistic regression, univariate screening of factors was performed firstly. The odds ratio (OR) values were calculated separately and the variables with *p* < 0.05 were included in the multivariate model. Using manual stepwise regression method, according to Akaike criteria, the likelihood ratio test (LRT) was used to screen variables and determine the final model. To further test the effectiveness of the model, the receiver operating characteristic (ROC) curve was used and the area under the ROC curve (AUC) was calculated. *p-*value < 0.05 was considered significant.

## Results

### Generation of a monoclonal antibody targeting the Gas6/AXL complex

To generate monoclonal antibodies that specifically recognizes the Gas6/AXL complex, we expressed human AXL (hAXL) in the mouse L929 cell line and subsequently mixed the cells with the Gas6-his fusion protein. Mice were then immunized with the pre-treated or untreated cells, respectively. After four rounds of immunization, splenocytes from the immunized mice were isolated and fused with SP2/0 cells. The supernatants from the hybridomas were characterized for their binding to AXL expressing cells pre-treated or untreated with Gas6-his fusion protein. Finally, three presentive clones of AXL antibody were obtained, named as αAXL-4#, αAXL-9# and αAXL-13#, respectively.

**Figure 1:**
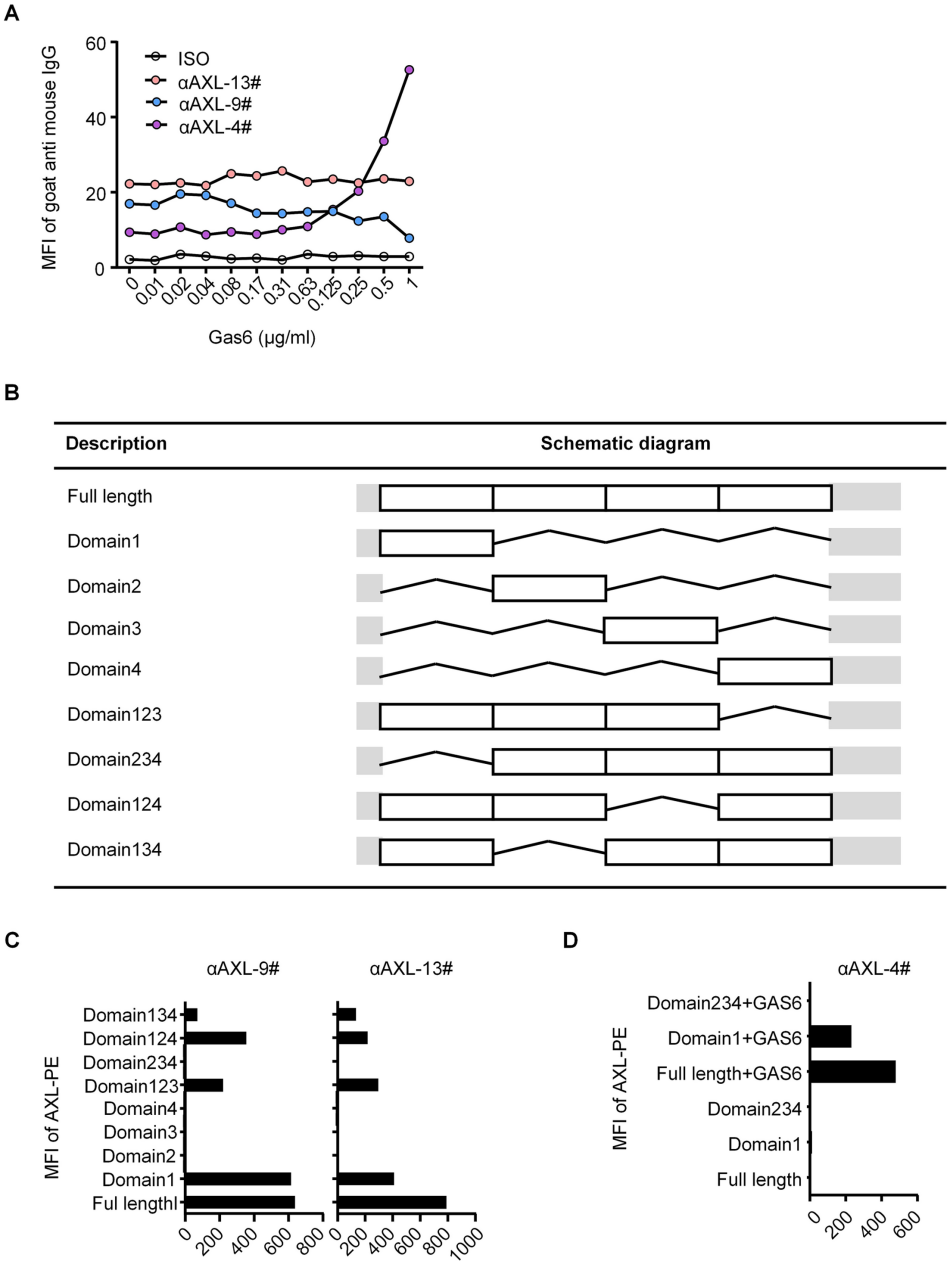
**(A)** Binding of different antibodies to AXL. Binding of different antibodies to AXL and their interaction with Gas6. **(B)** List of full length and different truncations of AXL. **(C)** αAXL-9# and αAXL-13# were analyzed via flow cytometry for reactivity towards hAXL or hAXL truncations. **(D)** αAXL-4# were analyzed via flow cytometry for reactivity towards hAXL or hAXL truncations with or without Gas6-his.

To assess the binding ability, we stained HEK293T cells overexpressing hAXL (293T-hAXL cells) with different concentrations of Gas6 fusion protein. Interestingly, αAXL-4# did not bind to 293T-hAXL cells in the absence of Gas6, but as the concentration of Gas6 increased, the mean fluorescence intensity (MFI) also rose gradually, indicating that the binding of αAXL-4# to the AXL protein is dependent on Gas6 binding. Conversely, the binding of αAXL-9# was competitively inhibited by Gas6, meaning the binding site or structure of αAXL-9# was affected by Gas6. However, for αAXL-13#, its binding was not associated with Gas6 (Figure 1A). According to whether AXL binding is affected by Gas6, these three clones of antibody were classified into three groups: dependent (αAXL-4#), competitive (αAXL-9#) and non-competitive (αAXL-13#). In particular, it was for this first time that a novel antibody specifically against Gas6/AXL complex was reported.

### Binding region mapping for AXL antibodies

**Figure 2:**
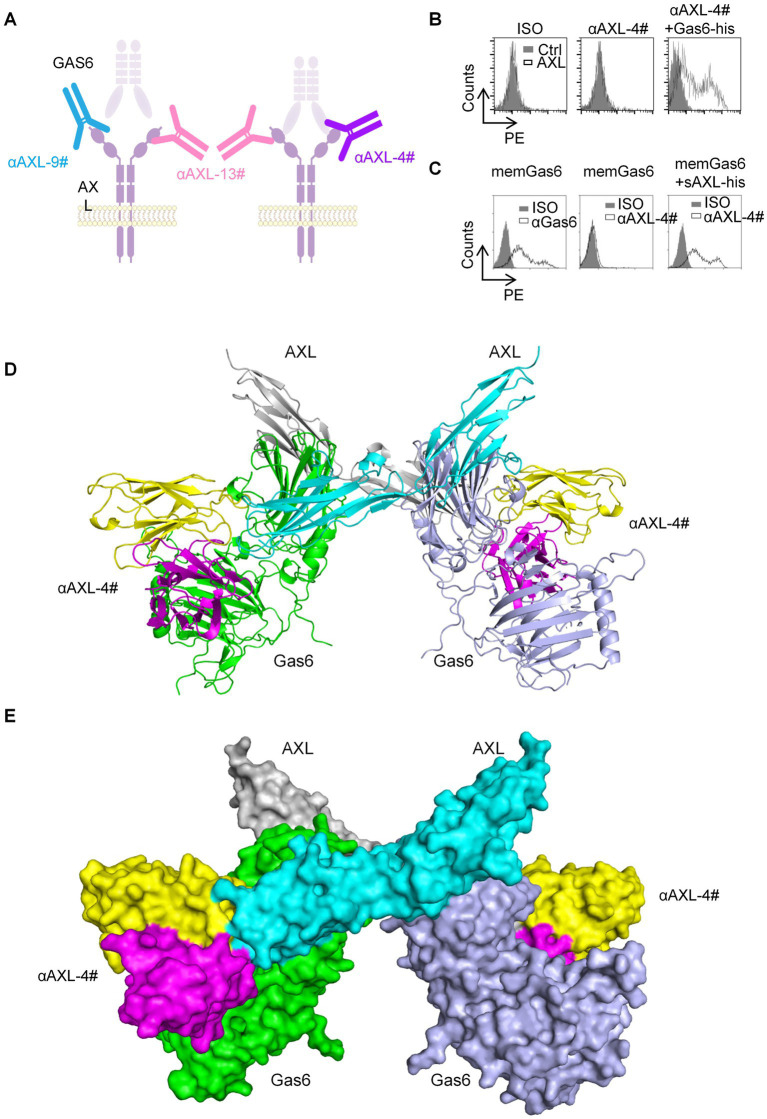
The binding characteristic of AXL-4#. **(A)** Representation of interplay of AXL antibody and Gas6. **(B)** 293T-hAXL cells were incubated with isotype control (left), AXL-4# (middle) or AXL4# with Gas6 (right), then stained with PE-goat anti mice IgG2a. **(C)** 293T-memGas6 cells were incubated with anti-Gas6l (left), AXL-4# (middle) or AXL4# with Gas6 (right), then stained with PE-goat anti mice IgG2a. **(D,E)** The structure of αAXL-4# recognizing Gas6/AXL complex predicted by AlphaFold.

As previously reported, Gas6 binds to the first immunoglobulin (Ig)-like domain of AXL (19). To analyze the molecular basis of these antibodies binding to AXL, we performed systematic mutagenesis and flow cytometric to delineate the specific regions of AXL. We constructed truncations that either included or excluded each of the outer membrane domains of AXL (Figure 1B). As shown in Figure 1C, αAXL-9# and αAXL-13# could bind to AXL expressing domain134, 124, 123, and domain1, but not to domain 234, 2,3 or domain 4, indicating that αAXL-9# and αAXL-13# bind to AXL depending on domain1. Similar as αAXL-9# and αAXL-13#, we confirmed that αAXL-4# could bind to 293 T-hAXL cells expressing both the full-length AXL and the first Ig-like domain in the presence of GAS6. However, αAXL-4# binding to AXL was abrogated by the deletion of the first Ig-like domain of AXL (Figure 1D). Consistent with previous results (Figure 1A), none of the truncated AXL constructs could bind to αAXL-4# in the absence of Gas6 (Figure 1D). Taken together, these data indicate that αAXL-4# recognizes both the first Ig-like domain of AXL and Gas6, as illustrated in Figure 2A.

As known, Gas6 binding to AXL lead to the formation of a minimal 2: 2 Gas6/AXL complex (19). Furthermore, we employed artificial intelligence-based approaches to predict and analyze the interaction structures of αAXL-4# and Gas6/AXL complex. The binding site of αAXL-4 includes the specific structures of both AXL and Gas6 (Figures 2D,E), which also explained the uniqueness of αAXL-4.

### αAXL-4# could specifically bind to Gas6/AXL complex expressed on cell membrane

Firstly, we constructed two types of HEK293T cells overexpressing hAXL (293T-hAXL cells) or hGas6 (293T-hGas6 cells) on their cell membrane. αAXL-4# could not bind to 293T-hAXL cells in the absence of Gas6 (Figure 2B, middle), but after pretreated with Gas6-his fusion protein, 293T-hAXL cells could be recognized with αAXL-4# (Figure 2B, right). Gas6 expressed on 293 T-hGas6 cells membrane (Figure 2C, left), when only added αAXL-4#, it could not bind to memGas6 (Figure 2C, middle). Only if additional AXL-his protein existed, αAXL-4# bind to 293T-hGas6 cells (Figure 2C, right). Altogether, αAXL-4# was confirmed to recognize the Gas6/AXL complex, but not AXL or Gas6 alone.

### Species specificity of αAXL-4# and its binding to plasmacytoid dendritic cells and other cell lines

**Figure 3:**
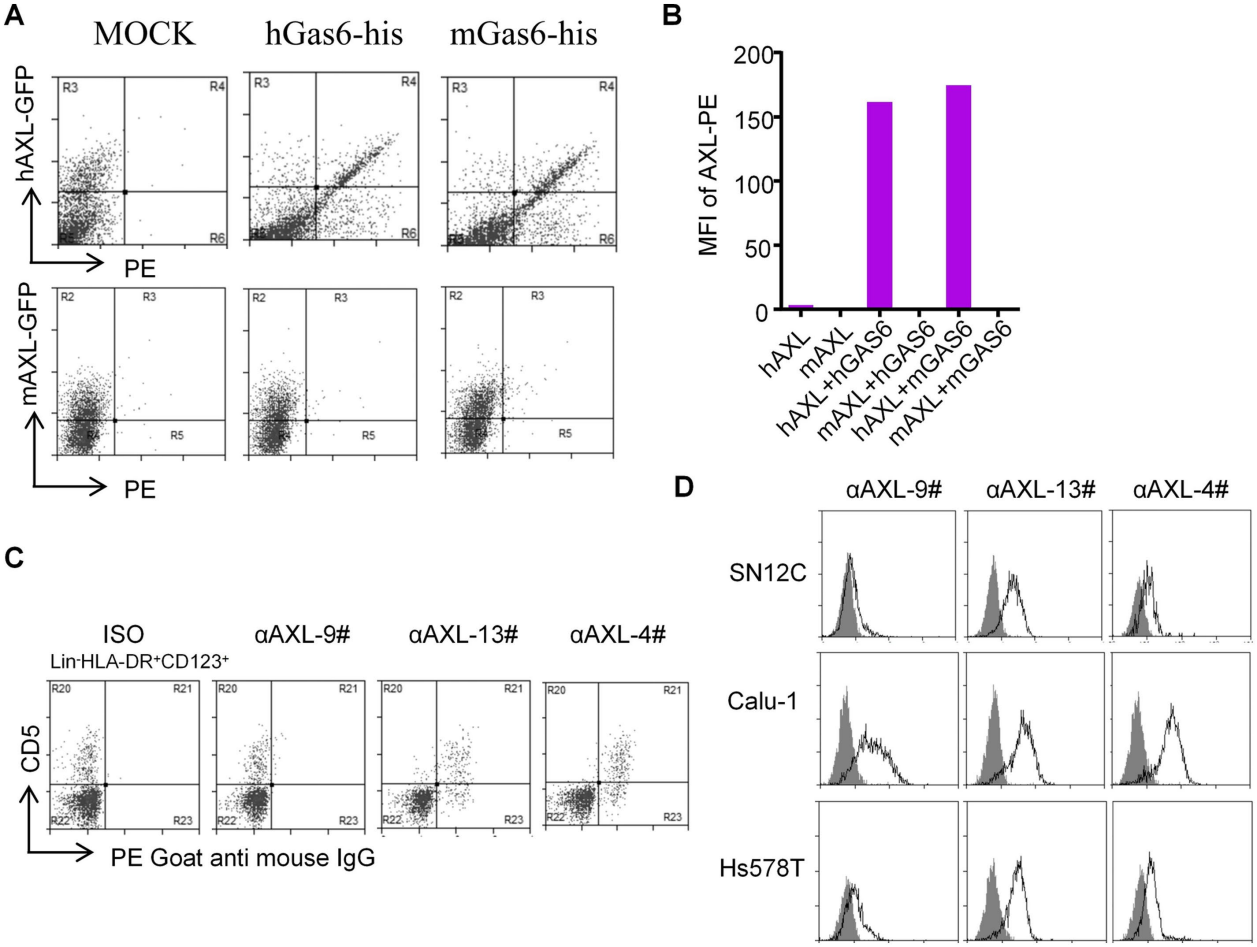
Application of αAXL-4# in flow cytometry. **(A,B)** The binding of αAXL-4# to Gas6/AXL complex of different Species. **(C,D)** asDCs and other cell lines were incubated with αAXL-9#, αAXL-13 and αAXL-4#.

Next, 293T-hAXL cells and HEK293T cells overexpressing mouse AXL (293T-mAXL cells) were used to test the species specificity of αAXL-4#. For 293T expressing human AXL, αAXL-4# could bind to Gas6/AXL complex containing human Gas6 or mouse Gas6, but it failed to bind to the complex consist of mouse AXL, indicating that there was no cross reaction between αAXL-4# and mouse Gas6/AXL complex (Figures 3A,B).

As reported, AXL is highly expressed on a newly identified subset of dendritic cells (DCs) in human, referred to as asDCs (20), which are also variably termed transitional DCs (tDCs), AXL^+^ DCs, and preDCs (21, 22), but it remains unclear whether AXL on its cell membrane has bind to Gas6. To solve this issue, we analyzed CD5^+^ asDCs within Lin^−^HLA-DR^+^CD123^+^ DCs derived from human peripheral blood mononuclear cells (23), and used αAXL-9#, αAXL-13# and αAXL-4# to bind AXL or Gas6/AXL complex, respectively. As expected, αAXL-13# could bind to pDCs. Interestingly, αAXL-4# also bound to asDCs (Figure 3C), while αAXL-9# did not. Hence, AXL expressed on human asDCs pre-binds to Gas6. Moreover, AXL is also highly expressed on various tumors and is often associated with poor prognosis (24). However, it remains unclear whether the forms of AXL present on tumor cells differ and which form contributes to tumor growth. To investigate this, we further analyzed the expression of AXL on several tumor cell lines using these three antibodies. For Calu-1 and Hs578T, AXL was partially bind to Gas6; however, in SN12C, AXL primarily exists as a Gas6/AXL complex (Figure 3D). The variations in the form of AXL indicate its potential to fulfill distinct roles within the AXL signaling pathway in corresponding cell types.

### ELISA assay for detecting soluble Gas6/AXL complex with αAXL-4#

**Figure 4:**
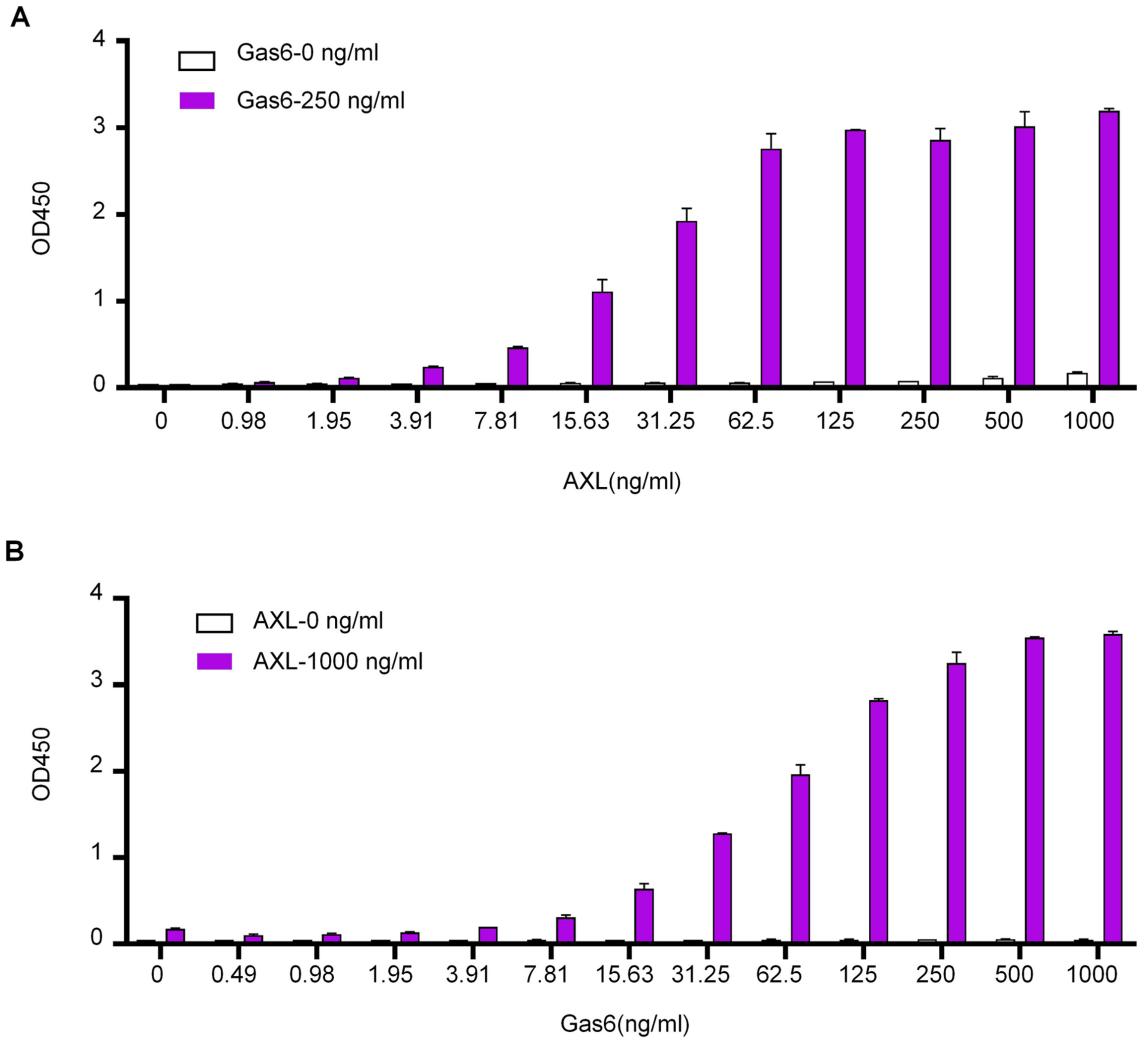
Application of αAXL-4# in ELISA. **(A)** Different concentrations of AXL (0-1 μg/mL) with a single dose of Gas6 (0.25 μg/mL, red column) or without Gas6 (empty column) were added subsequently. **(B)** Different concentrations of Gas6 (0–1 μg/mL) with a single dose of AXL (1 μg/mL, red column) or without AXL (empty column) were added subsequently.

The αAXL-4# antibody demonstrates the ability to bind to the Gas6/AXL complex expressed on the cell membrane. However, its potential interaction with the soluble Gas6/AXL complex, which is essential for conducting an ELISA detection assay, requires further investigation. To explore this, ELISA plates were coated with αAXL-4# as the capture antibody. Following a blocking step with BSA, varying concentrations of the soluble AXL-his fusion protein (sAXL) with or without the Gas6-his fusion protein were added. The complex was subsequently detected using a biotin-labeled AXL antibody that recognizes the first fibronectin type III (FN III)-like domain in a sandwich ELISA format. In the absence of Gas6, AXL was undetectable; conversely, in the presence of Gas6, the optical density (OD) values exhibited a positive correlation with increasing concentrations of AXL (Figure 4A). Additionally, when different concentrations of Gas6 were tested with or without sAXL, the results were consistent with those shown in Figure 3A (Figure 4B). We also determined that the linear detection range for AXL was found to be from 8 ng/mL to 62.5 ng/mL in the presence of 250 ng/mL Gas6, while the linear detection range for Gas6 was determined to be from 8 ng/mL to 250 ng/mL in the presence of 1,000 ng/mL sAXL. Collectively, these findings indicate that αAXL-4# can serve as a capture antibody for the quantitative determination of the soluble Gas6/AXL complex via ELISA.

### Dynamics of sAXL and Gas6/AXL complex in ACS patients

**Figure 5:**
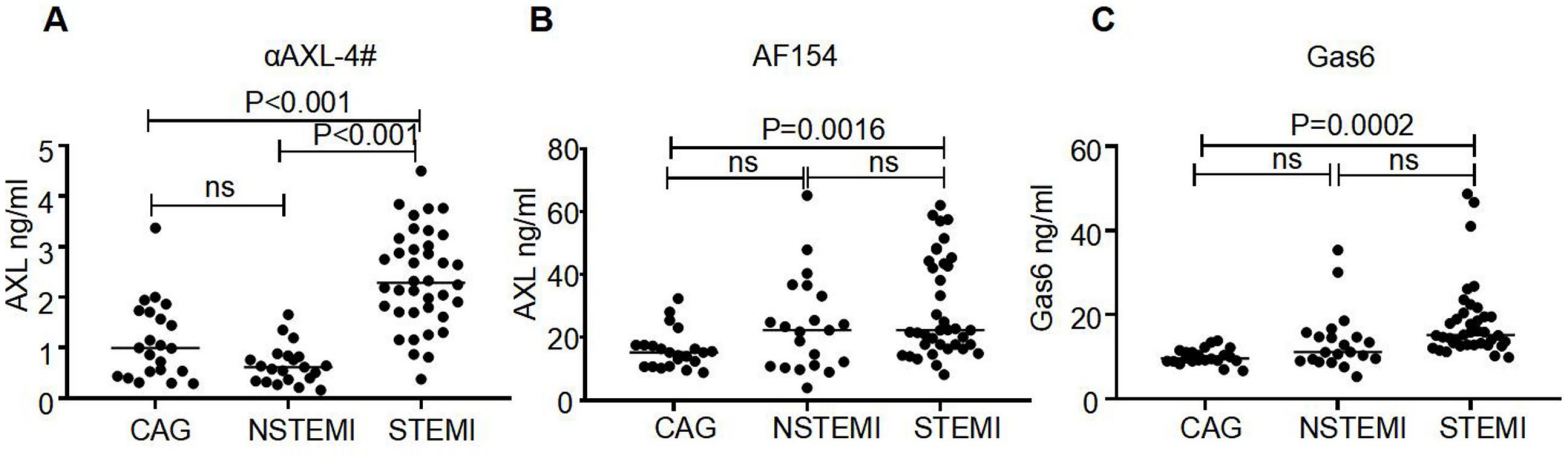
Different levels of AXL-Gas6, AXL and Gas6 in human plasma. **(A–C)** Plates were coated by AXL-4# **(A)**, commercial AXL antibody **(B)** or commercial Gas6 antibody **(C)** respectively. The levels of AXL-Gas6, AXL, or Gas6 in human plasma were detected by ELISA.

To investigate the dynamics of the two distinct forms of sAXL in CVD, we analyzed the levels of sAXL and the Gas6/AXL complex in patients with acute coronary syndrome (ACS). Baseline data were in Table 1. Utilizing a commercial ELISA kit (R&D), we observed that the level of sAXL was elevated in both ST-elevation myocardial infarction (STEMI) and non-ST-elevation myocardial infarction (NSTEMI) patients (Figure 5A), which is consistent with previous reports (10). However, no significant difference was noted between the STEMI and NSTEMI cohorts. Importantly, we found for the first time that the level of the Gas6/AXL complex significantly increased only in STEMI patients, while levels in NSTEMI patients remained unchanged. The complexed sAXL in STEMI patients exhibited approximately a three-fold increase compared to NSTEMI patients. The dynamics of the Gas6/AXL complex differ markedly from those of sAXL in these patient groups, indicating different clinical implications (Figure 5B). Additionally, we evaluated changes in Gas6, which was primarily elevated in STEMI patients, aligning with the findings for the Gas6/AXL complex (Figure 5C). Collectively, by employing αAXL-4# as a capture antibody, we were able to detect the dynamics of the two forms of sAXL in CVD and identify their distinct dynamics across different disease states.

### Elevated sAXL-Gas6 complex is a risk factor for STEMI

To further evaluate the clinical value of sAXL-Gas6, univariate and multivariate stepwise regression analyses were performed to assess the factors related to STEMI as dependent variables. The results of the logistic regression model are presented in Table 2. Univariate analysis revealed that, the Gas6/AXL complex, sAXL, Gas6, hyperlipidemia, left ventricular ejection fraction (LVEF), white blood cells (WBC), neutrophil percentage, blood glucose, alanine transaminase (ALT), aspartate transaminase (AST), low_density lipoprotein (LDL), high density lipoprotein (HDL) and brain natriuretic peptide (BNP) were significantly associated with STEMI (*p* < 0.05), while other factors were not statistically significant (*p* > 0.05). Multivariate logistic regression analysis indicated that STEMI was associated with the Gas6/AXL complex, hyperlipidemia, and LVEF. Specifically, hyperlipidemia and Gas6/AXL complex levels were positively correlated, and identified as risk factors, with odds ratios (OR) of 157.045 (7.171–3439.520, *p* < 0.001) and 3.307 (1.209–9.051, *p* = 0.020), respectively. Conversely, LVEF exhibited a negative correlation with an OR of 0.660 (0.508–0.856, *p* = 0.002).

**Table 2:** Logistic regression analysis with clinical characteristics as independent variable in AMI patients.

Variable	Univariate analysis				Multivariate analysis			
	Crude OR	95%CI		*p*	Adjusted OR	95%CI		*p*
AXL-Gas6 complex	3.036	1.599	5.761	0.001	3.307	1.209	9.051	0.020
sAXL	1.093	1.028	1.161	0.004				
Gas6	1.396	1.139	1.711	0.001				
Age	0.988	0.945	1.033	0.596				
Sex	0.484	0.159	1.469	0.200				
BMI	1.049	0.902	1.219	0.536				
Hyperlipidemia	25.667	5.194	126.836	0.000	157.045	7.171	3439.520	0.001
Smoke	2.500	0.883	7.078	0.084				
HBP	1.508	0.536	4.238	0.436				
Diabetes	0.406	0.135	1.221	0.109				
CHD family history	0.739	0.264	2.069	0.564				
LVEF	0.720	0.603	0.860	0.000	0.660	0.508	0.856	0.002
LVEDD	1.004	0.887	1.136	0.950				
WBC	2.173	1.470	3.212	0.000				
Neutrophil%	1.275	1.131	1.437	0.000				
RBC	1.058	0.416	2.692	0.906				
HGB	0.988	0.960	1.018	0.437				
PLT	1.002	0.994	1.010	0.615				
HCT	0.964	0.868	1.070	0.487				
Blood glucose	2.052	1.328	3.171	0.001				
HbA1c	1.356	0.845	2.173	0.207				
ALT	1.044	1.014	1.076	0.004				
AST	1.055	1.011	1.100	0.014				
LDL	2.933	1.285	6.693	0.011				
HDL	0.106	0.012	0.981	0.048				
TG	0.827	0.490	1.395	0.476				
TC	1.475	0.863	2.519	0.155				
BNP	1.004	1.000	1.008	0.040				
Cr	1.006	0.982	1.030	0.638				
BUN	0.912	0.681	1.221	0.537				
UA	0.998	0.994	1.002	0.429				
HCY	0.997	0.974	1.021	0.835				
CRP	0.996	0.970	1.024	0.801				
hsCRP	1.029	0.927	1.142	0.592				

### Association of Gas6/AXL complex with clinical data in STEMI patients

**Figure 6:**
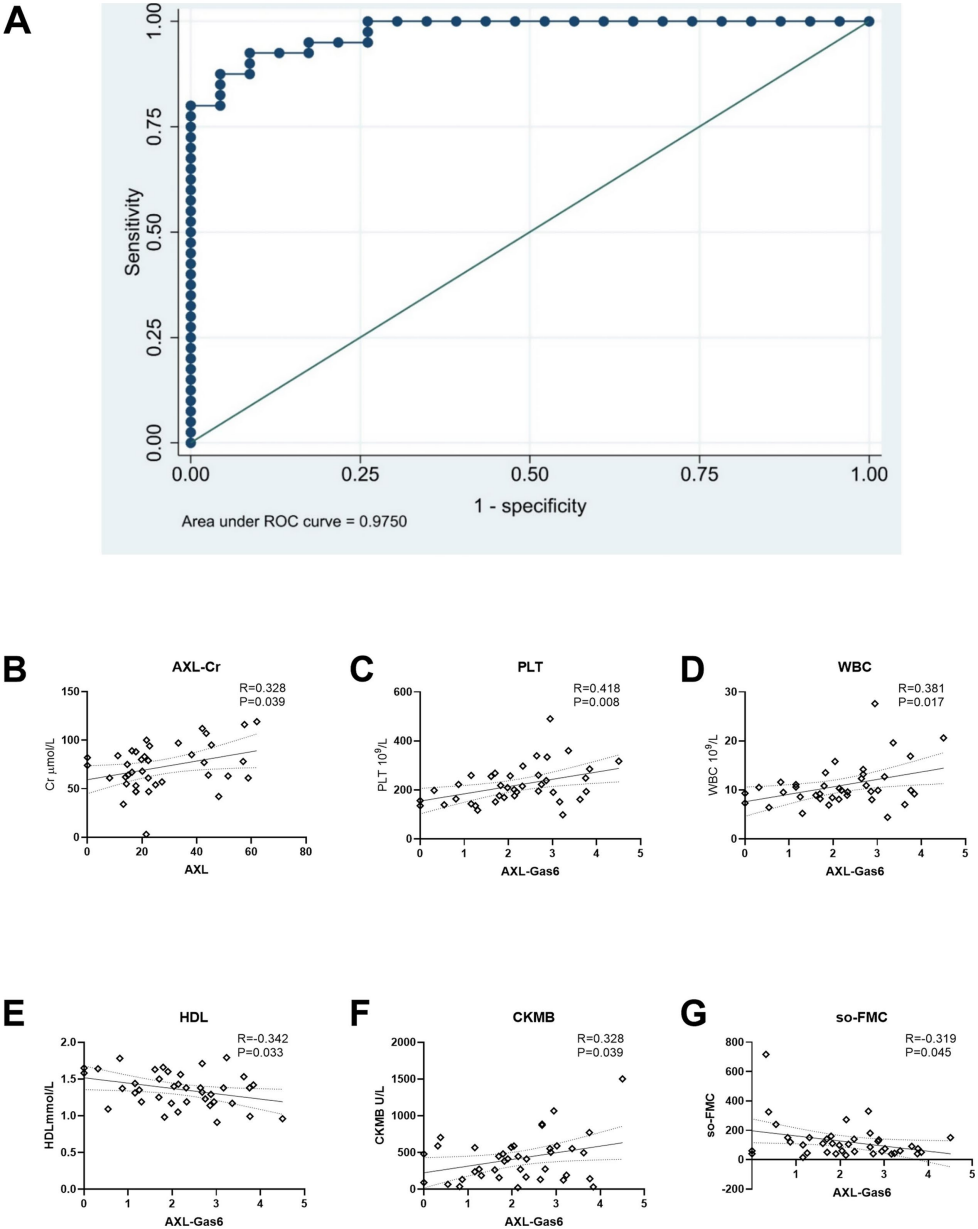
ROC curve and association with AXL/AXL-Gas6 with clinical data. **(A)** ROC curve; **(B)** AXL was associated with Cr in STEMI; **(C–G)** Gas6/AXL complex was associated with PLT, WBC, HDL, CK-MB and SO-FMC as indicated.

The Receiver Operating Characteristic (ROC) curve and its area were utilized to verify the effectiveness of the model (Figure 6A). Based on the ROC curve analysis of the constructed model, the Under the Curve (AUC) value was recorded at 97.50%, indicating that the model’s performance was acceptable according to AUC criteria. Furthermore, the model exhibited a sensitivity of 92.50% and a specificity of 91.30%, demonstrating its reliability. The threshold value for the Gas6/AXL complex was determined to be 1.8 ng/mL; concentrations exceeding this threshold were associated with an increased risk of myocardial infarction.

Additionally, we analyzed the correlation between the Gas6/AXL complex or sAXL and clinical data in STEMI patients. The levels of sAXL were found to positively correlated with creatinine levels (Figure 6B). In contrast, the Gas6/AXL complex exhibited positive correlations with creatine kinase MB (CM-KB), white blood cells (WBC) and platelets (PLT), while showing negative correlations with symptom onset-to-first medical contact (SO-to-FMC) and high-density lipoprotein (HDL) (Figures 6C–G). These findings suggest that, unlike sAXL, the Gas6/AXL complex is more valuable in assessing infarct size and the severity of myocardial infarction.

## Discussion

Recently a growing number of studies have confirmed that sAXL is an effective clinical biomarker in various diseases (25–28), not limited to cardiovascular disorders (9, 29). Since the Gas6/AXL complex in human blood was first reported in 2010 (11), its functions remain unclear. To date, a commercial kit for detecting the Gas6/AXL complex is still unavailable. Consequently, the dynamic changes of the Gas6/AXL complex under different pathological conditions are not well understood. To address this urgent issue, we generated a monoclonal antibody that specifically recognizes the Gas6/AXL complex at both the FACS and ELISA levels, which can serve as a capture antibody, enabling the detection of the soluble Gas6/AXL complex in human blood.

For the first time, we report that the Gas6/AXL complex is significantly elevated in patients with STEMI patients, and this elevation is more sensitive than either sAXL or Gas6 detected by conventional methods. Additionally, the Gas6/AXL complex, along with hyperlipidemia, serves as a risk factor for STEMI. In contrast to the levels of sAXL measured by a commercial kit, the Gas6/AXL complex shows a stronger association with CK-MB and SO-FMC, indicating that the Gas6/AXL complex, to some extent, reflects the size of the infarction. The ROC analysis demonstrates favorable model performance with balanced sensitivity and specificity, indicating discriminative power for myocardial infarction risk prediction which highlight the potential clinical applications.

This article is merely a retrospective study, aiming to further confirm the feasibility of the Gas6/AXL complex detection and preliminarily explore the potential clinical application value of the Gas6/AXL complex. Interesting, although the sample size of this study was limited, the levels of the Gas6/AXL complex in patients with STEMI were significantly higher than those in the control group.

In addition to myocardial infarction, a clinical trial has demonstrated that sAXL concentrations play a crucial role in predicting clinical outcomes for heart failure patients (9, 29). Furthermore, Gas6 levels have been associated with an increased risk of all-cause and cardiovascular mortality in patients experiencing acute heart failure (30). The significance of the Gas6/AXL complex in heart failure warrants further investigation.

In this study, we present the first report of a unique antibody validated though flow cytometry and ELISA. Utilizing this novel antibody, we discovered for the first time that the Gas6/AXL complex is significantly elevated in the plasma of STMEI patients. Compared to conventional methods for detecting sAXL, our measurement of the Gas6/AXL complex demonstrates greater sensitively and clinical relevance, thereby providing a more accurate reflection of severity in STEMI patients. Importantly, this study serves as a preliminary exploration of the implications of the Gas6/AXL complex, indicating the need for further relevant investigations beyond cardiovascular disorders.

## Data Availability

The data supporting this study are available from the corresponding authors upon request. All reagents and antibodies will be made available to members of the research community upon reasonable request after completion of a material transfer agreement.

## Supplementary material

SUPPLEMENTARY FIGURE S1 The ELISA standard curve. Mix Gas6 (250 ng/mL) with AXL (30 ng/mL) in a total volume at 20 μL for 1 h to obtain the AXL-Gas6 complex. Plates were coated with αAXL-4# antibody overnight at 4°C. After blocked, plates were incubated with plasma or standard protein (500, 250, 125, 62.5, 31.25, 15.625, and 0 pg/ml) for 2 h at room temperature. Detection antibody, streptavidin-HRP, substrate solution and stop solution were added in sequence. A microplate reader was used to determine the optical density (OD) at 45.
