# Supplementary figures and images for: Detection of Gas6/AXL complex and its expression changes in patients with ST-segment elevation myocardial infarction
Source: Front Med (Lausanne). 2025 Aug 26;12:1653708. doi: 10.3389/fmed.2025.1653708 (PMC12417524; doi:10.3389/fmed.2025.1653708)

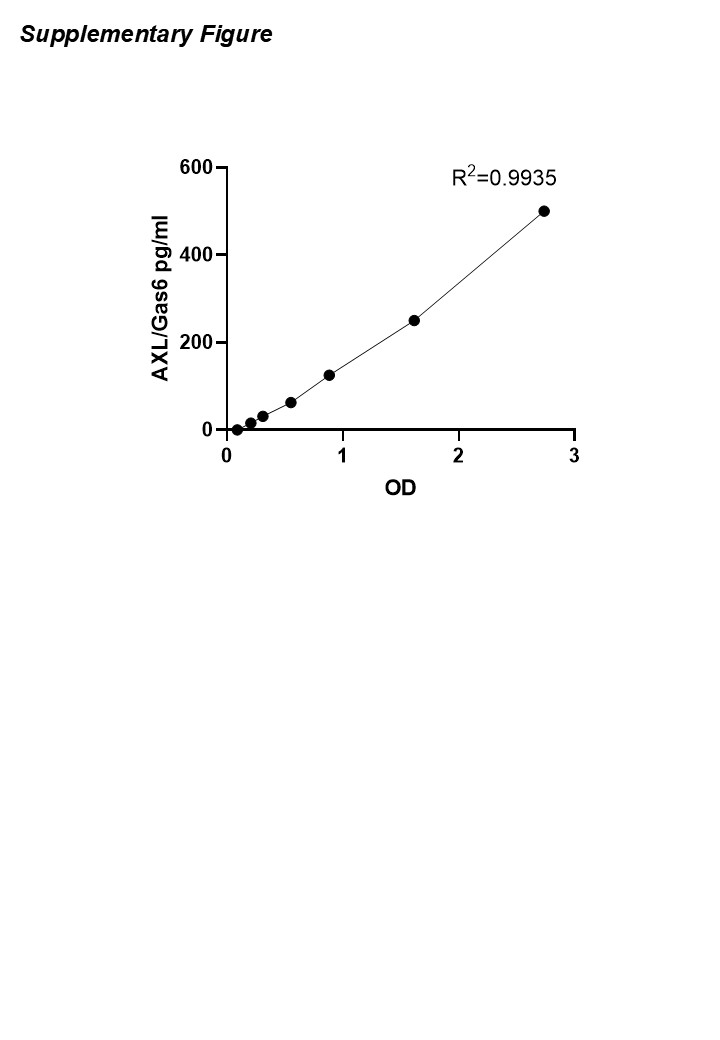

Supplement: SUPPLEMENTARY FIGURE S1 — The ELISA standard curve. Mix Gas6 (250 ng/mL) with AXL (30 ng/mL) in a total volume at 20 μL for 1 h to obtain the AXL-Gas6 complex. Plates were coated with αAXL-4# antibody overnight at 4°C. After blocked, plates were incubated with plasma or standard protein (500, 250, 125, 62.5, 31.25, 15.625, and 0 pg/ml) for 2 h at room temperature. Detection antibody, streptavidin-HRP, substrate solution and stop solution were added in sequence. A microplate reader was used to determine the optical density (OD) at 45. [file Image_1.JPEG]
